# AP-2*α* Inhibits c-MYC Induced Oxidative Stress and Apoptosis in HaCaT Human Keratinocytes

**DOI:** 10.1155/2009/780874

**Published:** 2009-12-24

**Authors:** Lei Yu, Michael J. Hitchler, Wenqing Sun, Ehab H. Sarsour, Prabhat C. Goswami, Aloysius J. Klingelhutz, Frederick E. Domann

**Affiliations:** ^1^Free Radical & Radiation Biology Graduate Program, Radiation Oncology Department, Roy J. and Lucille A. Carver College of Medicine, The University of Iowa, Iowa City, IA 52242, USA; ^2^Department of Microbiology, Roy J. and Lucille A. Carver College of Medicine, The University of Iowa, Iowa City, IA 52242, USA

## Abstract

AP-2*α* and c-MYC are important transcription factors involved in multiple cellular processes. They each display the paradoxical capacities to stimulate both cell proliferation and apoptosis under different conditions. In the present study we found that over expression of c-MYC was associated with accumulation of reactive oxygen species (ROS) and apoptosis in human keratinocytes, both of which were significantly inhibited by co-expression of AP-2. The effects of AP-2 on c-MYC were active at several levels. First, AP-2 and c-MYC were confirmed to interact at the protein level as previously described. In addition, forced expression of AP-2 significantly decreased steady state levels of c-MYC mRNA and protein. These findings suggested that AP-2 may have a direct effect on the *c-myc* gene. Chromatin immunoprecipitation assays demonstrated that AP-2 proteins bound to a cluster of AP-2 binding sites located within a 2 kb upstream regulatory region of *c-myc* These results suggest that the negative regulation of AP-2 on c-MYC activity was achieved through binding of AP-2 protein to the *c-myc* gene. The effects of AP-2 on c-MYC induced ROS accumulation and apoptosis in epidermal keratinocytes are likely to play an important role in cell growth, differentiation and carcinogenesis of the skin.

## 1. Introduction

The c-MYC protein, which is encoded by *c-myc* gene, is a nuclear phosphoprotein. This protein belongs to the helix-loop-helix/leucine zipper (HLH/LZ) family of transcription factors (including c-MYC, N-MYC, L-MYC, S- and B-MYC) and recognizes E-box sequences that contain a central CAC(G/A)TG sequence [1]. Although its precise cellular functions remain enigmatic, it is clear that c-MYC plays a critical role at some level in cell proliferation and differentiation [2]. For example, induction of c-MYC is sufficient to drive quiescent cells into the cell cycle, while inhibition of c-MYC can block mitogenic signals and facilitate cell differentiation [3]. A well-known human disease involving c-MYC is human Burkitt's lymphoma [4], in which *c-myc* is translocated from chromosome 8 to one of three chromosomes that contain antibody-encoding genes where its transcription is activated by those strong lymphocyte specific promoters. Gene amplification is another mechanism that leads to overexpression of c-MYC [5]. More recently, the overexpression and deregulation of *c-myc* in many of other types of tumors have been demonstrated [6]. Unlike other proto-oncogene products, c-MYC levels in different tumor types range from lower than their normal cells of origin to dramatically increased [7]. So deregulation, not simply amplification, is generally considered as the key point of carcinogenesis caused by c-MYC. Besides promoting cell proliferation, c-MYC also shows the capacity to induce cell death [8, 9]. At high levels of expression, c-MYC cannot only sensitize cells to cell death, but also affect neighboring cells [10, 11]. The apoptosis induced by c-MYC has been shown to depend on signaling via FasL/Fas pathway in T cells [12], increased cellular free radical levels [13], and can be either p53 dependent or p53 independent [14]. 

The exact mechanisms through which c-MYC mediates its diverse effects on cell fate are unknown. One possibility is that c-MYC functions as a classical transcription factor in the formation of heterodimers with Max or Mad and binds to E box related elements [15]. Another is the interaction of c-MYC with other proteins which have been predominantly mapped to occur through its C-terminal region (CTR) and N-terminal region (NTR). Among the proteins identified in a direct interaction with c-MYC, transcription factor AP-2*α* (TFAP2A) [2, 16] has been shown to have a similar pattern of biological behavior as c-MYC. 

AP-2 is a transcription factor family that includes five members: AP-2*α*, AP-2*β*, AP-2*γ*, AP-2*δ*, and the recently identified AP-2*ε*. All AP-2 family members share a common protein structure that includes a DNA binding domain, a dimerization domain, and a transactivating domain [17]. These transcription factors regulate the expression of genes involved in a wide spectrum of important biological functions, including cell proliferation, differentiation and carcinogenesis. AP-2*α* has previously been shown to interact with the BR/HLH/LZ domain of c-MYC through C-terminal domains (amino acids 204–437) of AP-2*α* and block the DNA binding of c-MYC [16]. Similar to c-MYC, the function of AP-2*α* also has been found to be paradoxical. Some investigators found that AP-2*α* was a tumor suppressor [18, 19] which had the capacity to induce apoptosis, while others suggested that AP-2*α* could be a proto-oncogene [20, 21] which stimulated cell proliferation. 

Although it has been shown that AP-2*α* has a negative effect on c-MYC transcriptional activity in two *bona fide* target genes, prothymosin-alpha and ornithine decarboxylase [16], it is not clear whether AP-2*α* has the capability to inhibit c-MYC induced transformation or apoptosis. Besides the known protein-protein interaction, whether there are other levels of interaction or regulation between these two transcription factors remains unknown. 

Both AP-2*α* [22] and c-MYC [23] can be induced by UVA irradiation. Furthermore, of HaCaT cells exposed to chronic UVA irradiation showed increased resistance to further UVA induced apoptosis [24]. Although this study did not measure the AP-2*α* levels in those treated HaCaT cells, expression of the AP-2 target gene MMP9 was found to be significantly higher. We found in our own study that overexpression of AP-2*α* in HaCaT cells before UVA irradiation could significantly increase cell survival (Supplemental Figure 1). These data strongly suggested that increased AP-2*α* levels may protect cells from further UVA induced cell death, and inhibition of UVA-induced c-MYC expression could be one of the mechanisms behind the protection. 

To better understand the biological effects of the interaction between c-MYC and AP-2*α*, we first overexpressed c-MYC and monitored the generation of reactive oxygen species (ROS) and cell biology parameters including clonogenic survival and growth rates; then we co-overexpressed AP-2*α* to test the hypothesis that AP-2*α* can block at least some consequences of c-MYC overexpression in HaCaT human keratinocytes. AP-2dn, a transactivating domain truncated protein [20], was also overexpressed in HaCaT cells to test whether the transcriptional activity of AP-2 was necessary for any effects on c-MYC activity.

## 2. Results

### 2.1. Overexpression of c-MYC Increased Intercellular ROS Levels

**Figure 1:**
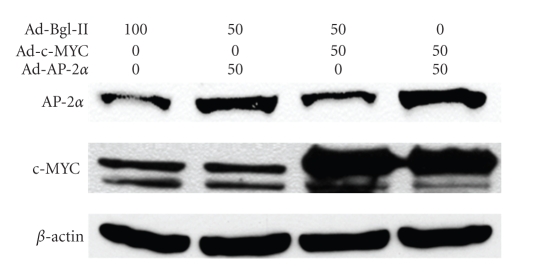
Adenovirus forced overexpression of AP-2 and c-MYC in HaCaT cells. HaCaT cells were infected with Ad-Bgl II 100 MOI (control), 50 MOI Ad-Bgl II + 50 MOI Ad-AP-2 (AP-2*α*), 50 MOI Ad-Bgl II + 50 MOI Ad-c-MYC (c-MYC), and 50 MOI Ad-AP-2 + 50 MOI Ad-c-MYC (AP-2*α* + c-MYC). On the c-MYC blot (middle) the immunoreactivity that corresponds to the forced overexpression of the c-MYC is shown by the upper band. The figure is a representative of at least three independent blots.

**Figure 2:**
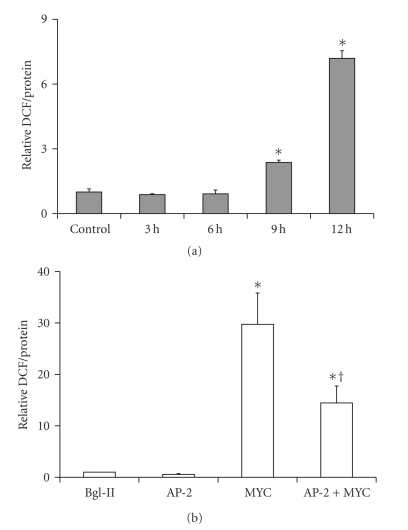
c-MYC induced accumulation of ROS in HaCaT cells was partially reversed by AP-2*α*. (a) time course showed that as early as 9 hours after infection, ROS level was higher than control (*P < .05 versus control, *n* = 3); (b) AP-2 reduced the ROS induced by c-MYC. The intercellular ROS level was measured by DCF method 24 hours after infection. Overexpression of AP-2 alone did not increase ROS level while co-overexpression AP-2 with c-MYC significantly decreased the accumulation of ROS (*P < .05 versus Bg-II and AP-2; ^†^ *P* < .05 versus MYC, *n* = 3).

Overexpression of c-MYC and AP-2*α* proteins in HaCaT cells was confirmed by western blot (Figure 1). As early as 9 hours after infection with 50 MOI Ad-c-MYC, the intercellular ROS level, as detected by DCF method, was significantly increased relative to controls (Figure 2(a)) and by 24 hours it reached to about a 30-fold increase (Figure 2(b)). Overexpression of AP-2*α* alone did not increase ROS level compared to control vector Bgl-II. Thus, c-MYC overexpression was linked to a significant increase of ROS, while co-overexpression of AP-2*α* with c-MYC alleviated the increase of ROS (Figure 2(b)). 

### 2.2. AP-2*α* Partially Prevented Cell Death Induced by Overexpression c-MYC in HaCaT Cells

**Figure 3:**
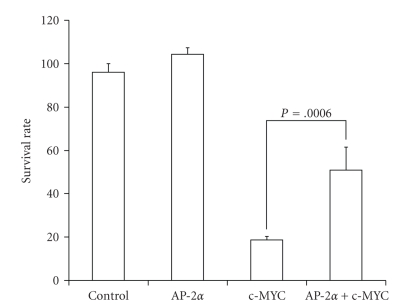
c-MYC induced cell death in HaCaT cells was partially reversed by AP-2*α*. Twenty-four hours after infection, 100 cells from each group were seeded and cultured under normal growth conditions for 10–14 days. Colonies with ≥50 cells were counted. AP-2*α* increased clonogenic survival in c-MYC overexpressing HaCaT cells. The surviving fractions were calculated by normalizing the number of colony of each treated group to that of the control group, respectively. Error bars stand for standard deviations, *n* = 3.

c-MYC overexpression caused a dramatic decrease of clonogenic cell survival, with surviving fractions decreasing to approximately 20% (Figure 3). Coexpression of AP-2*α* significantly rescued the cells and increased the surviving fraction, doubling it to approximately 40%. Forced AP-2*α* overexpression alone did not have a detectable effect on clonogenic survival in HaCaT cells at a titer of 50 MOI. This is in contrast to results previously determined for human breast carcinoma cells [25] and may reflect the relatively higher endogenous level of AP-2*α* expression in human keratinocytes.

### 2.3. AP-2*α* Attenuated c-MYC Induced Apoptosis in HaCaT Cells

**Figure 4:**
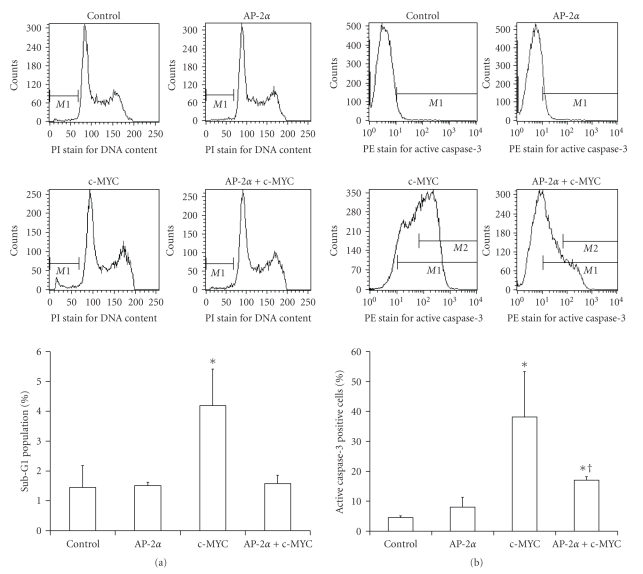
Wild type AP-2*α* attenuated apoptosis induced by c-MYC in HaCaT cells. HaCaT cells were infected with Ad-Bgl II 100 MOI (Control), 50 MOI Ad-Bgl II + 50 MOI Ad-AP-2 (AP-2*α*), 50 MOI Ad-Bgl II + 50 MOI Ad-c-MYC (c-MYC), or 50 MOI Ad-AP-2 + 50 MOI Ad-c-MYC (AP-2*α* + c-MYC). Propidium iodide (PI) staining for sub-G1 and phycoerythrin (PE)-conjugated anti-caspase-3 antibody staining for active caspase-3 were performed as indicated in the Materials and Methods section. (a) AP-2*α* decreased the sub-G1 population induced by c-MYC. Upper panels: representative flow cytometry plots of PI-sub-G1 DNA content (M1) in the variously treated cells. Lower panel: quantitative determination of sub-G1 DNA content. Error bars represent standard deviations, *n* ≥ 4. *P < .01 compared to control, AP-2*α*, and AP-2*α* + c-MYC groups. (b) AP-2*α* decreased the percentage of cells positive for active caspase-3 induced by c-MYC. Upper panels: representative flow cytometry plots of measurement of PE-active caspase-3. Lower panel: quantitative determination of active caspase-3. Error bars represent standard deviations, *n* ≥ 4. *P < .01 compared to control, ^†^ *P* < .05 compared to control and c-MYC group.

Two independent assays for apoptosis, propidium iodide (PI) DNA staining and caspase-3 activation, showed that overexpression of c-MYC in HaCaT cells induced apoptosis (Figure 4). Fifty MOI of Ad-c-MYC increased the sub-G1 apoptotic population about 3 times compared to the control group (*P* < .01) (Figure 4(a)), while coexpression of AP-2*α* relieved that increase back to the basal level. Although sub-G1 content DNA level by PI staining indicates death, it is not specific for apoptosis. Thus, the activated caspase-3 assay was employed as it is more specific to the detection of apoptosis in these cells (Figure 4(b)). c-MYC increased the fraction of the active caspase-3 positive population from 4.5% to 40% (~10 fold), while coexpression of AP-2*α* decreased this increase to around 15% (~3 fold) (Figure 4(b)).

### 2.4. AP-2*α* Inhibits the Expression of Endogenous c-myc

**Figure 5:**
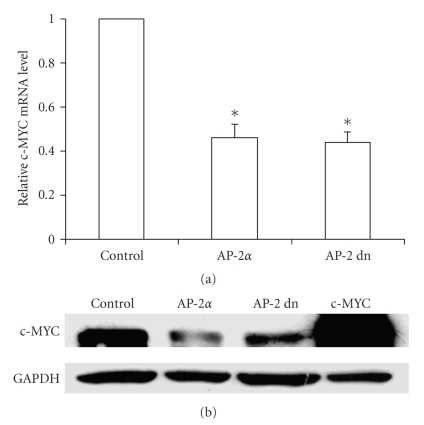
Both wild type and dominant negative AP-2 inhibit endogenous *c-myc* expression. HaCaT cells were infected with Ad-Bgl II (Control), wild type AP-2*α*, dominant negative AP-2, or c-MYC adenovirus at 100 MOI. Twenty-four hours later, nuclear protein and total RNA were extracted. (a) c-MYC mRNA level decreased to about 50% compared to control group after AP-2 infection (*P < .05 versus BglII control, *n* = 3). (b) c-MYC protein level decreased after AP-2 infection, The fourth lane is a positive control sample from Ad-c-MYC infected HaCaT cells (representative blot of three independent experiments).

**Figure 6:**
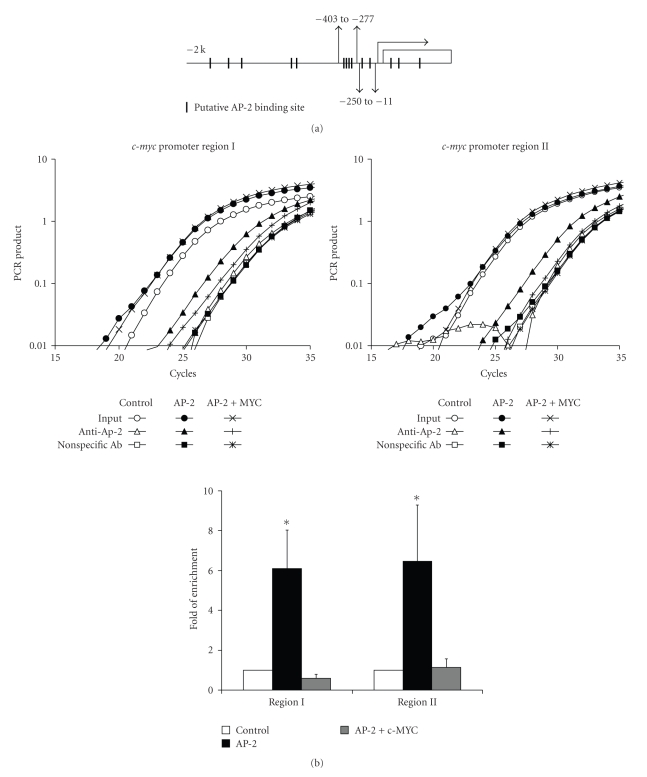
AP-2*α* protein bound to *c-myc *5′ flank regulatory region. (a) Diagram of the location of AP-2 putative binding sites in *c-myc *5′ flank regulatory region (short columns). The gray short arrow heads represent the primers for ChIP assay. (b) Overexpression of AP-2*α* caused enrichment of two promoter regions DNA with anti-AP-2 antibody in ChIP assay. Region I (−403 to −277) and Region II (−250 to −11) were both enriched about 6 folds. Cooverexpression of c-MYC (50 MOI) decreased AP-2 binding to *c-myc* promoter back to basal level. Upper panels are representative amplification curves of the real time PCR for ChIP assay; lower panel is a plot of the quantification of the data calculated with ΔΔCt. Error bars stand for standard deviation (SD), *n* = 3. *P < .05 compared to control.

After we found that overexpression of AP-2 could inhibit at least some of the consequences of exogenously expressed c-MYC, we further investigated whether AP-2 had similar effects on endogenous c-MYC. AP-2*α* and c-MYC are both transcription factors; therefore, besides the direct protein interaction, it is possible that these two factors affect each others' gene expression. We found that overexpression of AP-2*α* decreased the steady-state levels of both c-MYC mRNA and protein compared to the vector control (Figures 5(a) and 5(b)). Interestingly, a transactivation domain deletion mutant of AP-2*α* had a similar effect, suggesting that there is a blocking effect of AP-2 on the c-MYC promoter. Analysis of 2 kb 5′ flanking region of the *c-myc* gene revealed that there were 11 putative AP-2 binding sites located within that area and three more in the first intron (Figure 6(a)). To determine whether AP-2 is capable of direct DNA binding to these *cis* elements, we performed in vivo quantitative chromatin immunoprecipitate (qChIP) assays. The qChIP assays showed that overexpression of AP-2*α* caused significant enrichment (approximately 6 folds) for both selected promoter regions analyzed compared to the vector control group (Figure 6(b)). Another genomic region that was previously known to contain an AP-2 binding site, the ICAM-1 promoter [22], was used as a positive control, and was enriched approximately 10 folds (data not shown). Since the interaction between these two proteins required the C-terminal end of both proteins [16] and blocked c-MYC DNA binding, we tested whether this interaction also blocked AP-2 DNA binding. We found, *in vitro*, that an increase of c-MYC protein amount caused a decrease of AP-2 DNA binding which was detected by gel shift assay (Supplementary Figure 2 in supplementary material available online at doi 10.1155/2003/780874) and, *in vivo*, cooverexpression of c-MYC could decrease the binding of AP-2 to *c-myc* promoter (Figure 6(b)).

## 3. Discussion

The Myc family of nuclear oncoproteins, including c-MYC, N-MYC, and L-MYC are key cell growth regulators. Among them, c-MYC has attracted a great deal of attention because of its broad relationship to human oncogenesis. c-MYC is overexpressed in many cancer types while at the same time, it also exhibits the ability to induce apoptosis [6, 9]. The mechanisms through which c-MYC mediates its diverse effects are unknown. Besides functioning as a traditional transcription factor binding to DNA elements, c-MYC has been also found to directly interact with a diverse array of other proteins including p107, BIN1, TRRAP, MM-1, AMY-1, Pam, *α*-tubulin, Max, YY1, AP-2, TFII-I, BRCA1, and Miz-1 [2]. The C-terminal domain (CTD) of c-MYC interacts with Max to form heterodimers and binds to DNA elements, while the N-terminal domain shows both transcription activation and transcription repression functions. Gaubatz et al. showed that AP-2*α* interacted with the c-MYC CTD both directly and indirectly [16]. These authors showed that AP-2*α* inhibited c-MYC DNA binding activity at the ornithine decarboxylase promoter either by competing with Myc/Max heterodimers for the E-box at the promoter region or by interacting with c-MYC directly within its CTD. 

In the present study we found that overexpression of c-MYC in HaCaT human keratinocytes induced apoptosis and significantly decreased clonogenic survival, and that this was associated with significantly increased ROS levels. Since AP-2*α* has been shown to inhibit c-MYC activity possibly through a protein-protein interaction [16], we hypothesized that AP-2*α* attenuated apoptosis induced by c-MYC through this mechanism; and since the protein-protein interaction is via the C-terminus of AP-2*α*, we also hypothesized that the transactivating domain of AP-2*α* was not required for such inhibition. Our current findings, that co-overexpression of AP-2*α* resulted in decreased ROS levels, increased survival, and decreased apoptosis induced by c-MYC provide support for these hypotheses. More importantly, our study demonstrates for the first time that AP-2*α* directly bound to the *c-myc *5′ flanking regulatory region *in vivo* and blocked the expression of c-MYC mRNA (Figures 5 and 6). There are 11 putative AP-2 binding sites located within the 2 kb 5′ flanking region of the *c-myc* gene. We chose two clusters of these sites close to the transcription start site for ChIP analysis. We found that when AP-2 (either wild type or dominant negative) was overexpressed, c-MYC expression was inhibited and at the same time, the binding of AP-2 to those two clusters increased approximately by 6 folds. Analysis of the *c-myc* promoter sequence also revealed other transcription factor binding sites overlapping the AP-2 sites. These transcription factors include Sp1, NF-1, GATA-1, and GR. Since Sp1 mediated the activation of *c-myc* [26] as well as many other genes, the overlapping AP-2 binding sites suggest a mechanism for c-MYC inhibition similar to that by which AP-2*α* inhibits the expression of MnSOD by competing with Sp1 on the MnSOD promoter [21]. 

Both c-MYC and AP-2*α* proteins have paradoxical effects in carcinogenesis and apoptosis. In our study we found that at 50 MOI, Ad-c-MYC could cause a massive apoptosis in HaCaT cells. HaCaT cells express mutant p53 [27, 28], thus we believe that the apoptosis induced by c-MYC in our study was p53 independent. AP-2*α* has been shown to directly interact with p53 [29] and may thus help regain the DNA binding and transcriptional activity of mutant p53. The inhibition of the apoptosis executed by AP-2*α* in our study seems to occur through a direct block to c-MYC protein and was p53 independent. 

In summary, as a negative regulator for c-MYC, AP-2*α* inhibits c-MYC at two different levels. First, AP-2*α* directly inhibits *c-myc* gene expression through binding to its 5′ regulatory region. Second, AP-2*α* has the ability to attenuate the apoptosis induced by c-MYC through protein-protein interaction and this effect did not require the transcriptional activity of AP-2.

## 4. Materials and Methods

### 4.1. Adenovirus Construction

The AP-2dn cDNA (a mutant form of AP-2*α* with a deletion of the transactivation domain encompassing amino acids 31–117) was provided by Dr. Lubomir Turek and was subcloned into pcDNA3 [30]. The coding sequences were then cloned into a shuttle vector provided by Gene Transfer Vector Core, the University of Iowa. The E1A replication deficient adenovirus was constructed with the established protocol [31]. The wild type AP-2*α* [30] adenovirus was constructed with same method and has been previously described [25]. The Ad-c-MYC [32] was amplified and maintained by the Gene Transfer Vector Core at The University of Iowa.

### 4.2. Cell Culture

HaCaT cells [33] were cultured in Dulbecco's Modified Eagle Medium (DMEM) supplemented with 10% FBS at 37°C in a humidified atmosphere of 5%  CO_2_ and 95% air to reach 70–90% confluence prior to adenovirus infections. For clonogenic survival analyses, cells were infected with Ad-Bgl II, Ad-AP-2*α*, Ad-c-MYC, or Ad-AP-2dn as indicated. Twenty-four hours later, cells were trypsinized and counted. One hundred cells from each group were seeded into each well of six-well plates. After 14 days under normal growth condition, the resulting colonies were stained with 0.5% crystal violet in 70% ethanol, and colonies with ≥50 cells were counted under dissection microscope. For growth curve, cells in sample wells were counted every other day.

### 4.3. Intracellular Peroxide Measurement

The intracellular peroxide concentration was measured with 2′,7′-dichlorodihydrofluorescein diacetate (DCFH-DA) as previously described [34]. Briefly, cells were washed with 1 × PBS, and 3 mL of 10 *μ*M DCFH-DA (20 *μ*L of 20 mM stock in 40 mL PBS, Molecular Probes) was added to each plate and incubated at 37°C for 30 minutes. Cells were washed with PBS, and then lysed with 800 *μ*L of 0.5% NP-40. Cell lysates were centrifuged at 10 000 × g at 4°C for 3 minutes. 180 *μ*L of lysate from each sample was loaded into 96-well NUNC black bottom plate and read in a SPECTRAFLUOR Plus microplate reader (TECAN, Austria) with excitation wavelength of 485 nm and emission wavelength of 530 nm. The protein concentration in the remaining lysate was determined and DCF fluorescence intensity per mg protein was calculated. In separate control groups, an oxidized (DCF-DA) probe was used the same way to control for the uptake and efflux of the probe. 

### 4.4. Apoptosis Assay

Two methods were applied to analyze the fraction of apoptotic cells. For propidium iodide (PI) staining, 1 × 10^6^ cells from each group were fixed with 70% ethanol at 4°C for one hour. Cells were then washed two times with 1 × PBS and then stained with 50 *μ*g/mL PI at the presence of 0.25 mg/mL RNase A at 4°C for 30 minutes at dark. The Active Caspase-3 Apoptosis Kit (BD Pharmingen, catalog number 550914) was used for the active caspase-3 analysis according to the manufacturer's instructions. The number of cells in the sub-G1 population was obtained by counting 10 000 cells for each sample by flow cytometry (Becton Dickinson FACscan). For active capase-3, 30 000 cells were counted for each sample.

### 4.5. Nuclear Protein Extraction

After treatment, cells were harvested by trypsinization and collected by centrifugation in 15 mL tubes. Cell pellets from individual cultures were rinsed twice in ice-cold PBS and resuspended in 0.5 mL of ice-cold hypotonic buffer [20 mM HEPES-HCl (pH 7.6), 1 mM ethylenediamine tetraacetic acid (EDTA), 10 mM NaCl, 1 mM dithiothreitol, and 0.5% Nonidet P-40] containing protease inhibitors. The cell suspension was transferred to a 1.5  mL microfuge tube, incubated on ice for 20 minutes, vortexed for 10 seconds, and centrifuged for 60 seconds at 6 000 g. The supernatant (cytoplasmic extract) was transferred to a new tube. The pelleted nuclei were washed twice with 1 mL of ice-cold hypotonic buffer and resuspended in 0.1 mL of extraction buffer (20 mM HEPES-HCl pH 7.6, 1 mM EDTA, 430 mM NaCl, 1 mM dithiothreitol, and 0.5% Nonidet P-40) containing the protease inhibitors. After 20 minutes on ice with occasional shaking, the suspension was centrifuged for 15 minutes at 16 000 g at 4°C, and the supernatant (nuclear extract) was transferred to a new tube. After determination of protein concentration, aliquots of each extract were stored at −80°C until use.

### 4.6. Western Blot Assay

Proteins were boiled with same volume of 2 × sample buffer (125 mM Tris pH 6.8, 4% SDS, 10% glycerol, 0.006% bromophenol blue, and 1.8% beta-mercaptoethanol) and were separated in a 12% SDS-PAGE gel, then transferred to nitrocellulose membranes at 100 V, 4°C for 1 hour. The membrane was blocked with 5% nonfat milk at room temperature for 1 hour and incubated with either AP-2*α* antibody 3B5 (Santa Cruz Biotechnology, Santa Cruz, CA) or MYC antibody (obtained from University of Iowa Hybridoma Core) 1 : 1000 at room temperature for 1 hour. The membrane was washed with 1 × TBST (10 mM Tris-HCl, pH 7.5, 150 mM NaCl, 0.05% Tween-20) buffer 5 times for 5 minutes each and then incubated with the second antibody (goat anti-mouse, HRP labeled, 1 : 10 000) at room temperate for 45 minutes. The membrane was washed again with 1 × TBST buffer 5 times for 5 minutes each time and the proteins were visualized by adding ECL buffer to the membrane and exposing to X-ray film.

### 4.7. RNA Extraction, Reverse Transcription, and Quantitative PCR for Measuring c-MYC mRNA

RNeasy Kits (Qiagen) were used to extract total RNA following manufacturer's protocol. High Capacity cDNA Archive kits from ABI (Foster City, CA) were used to prepare cDNA following manufacturer's protocols. Briefly, 2 *μ*g of total RNA was reverse transcribed to cDNA in a 100 *μ*L reaction at 37°C for 2 hours. Every 1 *μ*L RT reaction is equivalent to cDNA resulting from 20 ng RNA. Steady state c-MYC mRNA levels were measured using the SYBR Green method (ABI, Valencia, CA) according to manufacturer's instructions. The primers for c-MYC were forward: 5′-CCA CAC ATC AGC ACA ACT ACG CT and reverse: 5′-GCA TTT TCG GTT GTT GCT GAT C. The primers for 18S were forward: 5′-ACC GCG GTT CTA TTT TGT TG and reverse: 5′-CCC TCT TAA TCA TGG CCT CA. The relative c-MYC mRNA levels were calculated by normalizing to 18 seconds and comparing the ΔΔCt between control group and AP-2 overexpressed group according to manufacturer's instructions. 

### 4.8. In Vivo Quantitative Chromatin Immunoprecipitation (qChIP) Assay

Twenty million HaCaT cells from each group were cross-linked for 15 minutes at 37°C using 1% formaldehyde. Cells were washed twice with 1 × PBS. The cells were then pelleted by centrifugation at 500 g for 10 minutes and resuspended in 500 mL sonication buffer (50 mM Tris-Cl pH 8.1, 10 mM EDTA, and 1% SDS + protease inhibitors) and sonicated twice for 10 seconds to achieve an optimal fragment length between 300–600 bp. Cross-linked DNA/proteins were then diluted 1 : 10 using IP dilution buffer (0.01% SDS, 1.1% Trition-X 100, 1.2 mM EDTA, 16.7 mM Tris-Cl, pH 8.1, 167 mM NaCl) plus protease inhibitors. Samples were then precleared using 70 *μ*L of Protein G agarose (Upstate Biotech, Charlottesville, VA) for 1 hour at 4°C. Approximately 1/10th of the sample was removed as input control, while the remainder was immunoprecipitated with 10 *μ*g of anti-AP-2*α* (Upstate Biotech, Charlottesville, VA) or control mouse IgG overnight at 4°C with agitation. Chromatin/antibody complexes were then collected using Protein G agarose followed by washing and elution according to the manufacturer's procedure. DNA was purified from input chromatin and immunoprecipitation elutions by reversing cross-links using 200 mM NaCl at 65°C for 4 hours followed by the Qiagen DNeasy Kit (Qiagen, Valencia, CA) according to manufacturer's protocol. Purified DNA was then quantified by using a Biophotometer (Eppendorf, Hamburg, Germany). Five ng of DNA from input, anti-AP-2 pull down, and nonspecific antibody pull down were analyzed with real time PCR from each of the following groups: total input, anti-AP-2 IP, and nonspecific antibody IP. 

The primer sequences for amplifying the *c-myc* promoter region I were forward: 5′-TCC ATG CGG CTC TCT TAC TCT G and reverse: 5′-GCT TTG GGA ACC CGG G. For the second region they were forward: 5′-ATC CTC TCT CGC TAA TCT CCG C and reverse: 5′-AAT TAC TAC AGC GAG TTA GAT AAA GC. We used a previously known AP-2 binding site in ICAM-1 promoter [22] as a positive control. The primer sequences for the ICAM-1 promoter region: forward: 5′-AGG ATG ACC CTC TCG GCC and reverse: 5′-TGC TGC AGT TAT TTC CGG ACT. The fold-enrichments were calculated by comparing the ΔΔCt [35] between control group and AP-2 overexpressed group. ΔCt = Ct (Anti-AP-2 pull down) − Ct(Nonspecific Ab pull down). ΔΔCt = ΔCt (AP-2 overexpressed group) − ΔCt (Control group). Fold of enrichment = 2^−ΔΔCt^.

### 4.9. Statistical Analysis

A single factor ANOVA, followed by Student's *t*-test was used to compare statistical differences between means. Statistical significance was set at the 0.05 level.

## Supplementary Material

Supplemental Figure 1. Overexpression of AP-2*α* increased HaCaT cell survival rate upon UVA irradiation. HaCaT cells were infected with 100 MOI Ad-Bgl II or Ad-AP-2*α* for 24 hours. The cells then received 5 J per cm^2^ UVA irradiation. 100 cells were seeded immediately after UVA irradiation per plate. Clones that contained more than 50 cells were counted 2 weeks later. The survival rate of Bgl II control group was set arbitrarily to 100%. Error bars stands for SD, n = 3.
Supplemental Figure 2. Overexpression c-MYC protein caused decreasing of AP-2*α* DNA binding activity. HaCaT cells were infected with increasing MOI of Ad-c-MYC. 24 hours later, the nuclear protein was extracted and 40 *μ*g was analyzed with western blot and 5 *μ*g was analyzed with gel shift assay. AP-2*α* DNA binding activity decreased while c-MYC protein levels increased. AP-2*α* protein level also showed no significant increased after c-MYC infection.
