# Supplementary material for: AP-2α Inhibits c-MYC Induced Oxidative Stress and Apoptosis in HaCaT Human Keratinocytes
Source: J Oncol. 2009 Dec 24;2009:780874. doi: 10.1155/2009/780874 (PMC2801504; doi:10.1155/2009/780874)
Supplement: Supplementary file 1 — Supplemental Figure 1. Overexpression of AP-2α increased HaCaT cell survival rate upon UVA irradiation. HaCaT cells were infected with 100 MOI Ad-Bgl II or Ad-AP-2α for 24 hours. The cells then received 5 J per cm2 UVA irradiation. 100 cells were seeded immediately after UVA irradiation per plate. Clones that contained more than 50 cells were counted 2 weeks later. The survival rate of Bgl II control group was set arbitrarily to 100%. Error bars stands for SD, n = 3. Supplemental Figure 2. Overexpression c-MYC protein caused decreasing of AP-2α DNA binding activity. HaCaT cells were infected with increasing MOI of Ad-c-MYC. 24 hours later, the nuclear protein was extracted and 40 μg was analyzed with western blot and 5 μg was analyzed with gel shift assay. AP-2α DNA binding activity decreased while c-MYC protein levels increased. AP-2α protein level also showed no significant increased after c-MYC infection. [file 780874.f1.pdf]

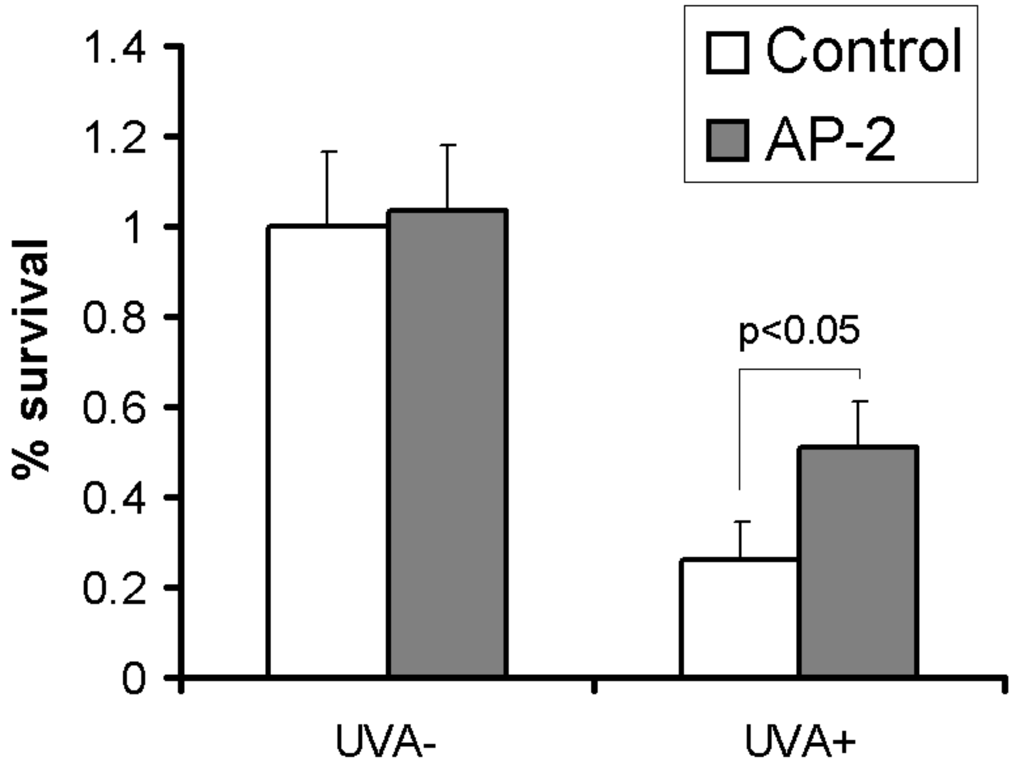

Supplemental Figure 1. Overexpression of AP-2α increased HaCaT cell survival rate upon UVA irradiation. HaCaT cells were infected with 100 MOI Ad-Bgl II or Ad-AP-2α for 24 hours. The cells then received 5 J per cm^2 UVA irradiation. 100 cells were seeded immediately after UVA irradiation per plate. Clones that contained more than 50 cells were counted 2 weeks later. The survival rate of Bgl II control group was set arbitrarily to 100%. Error bars stands for SD, n=3.

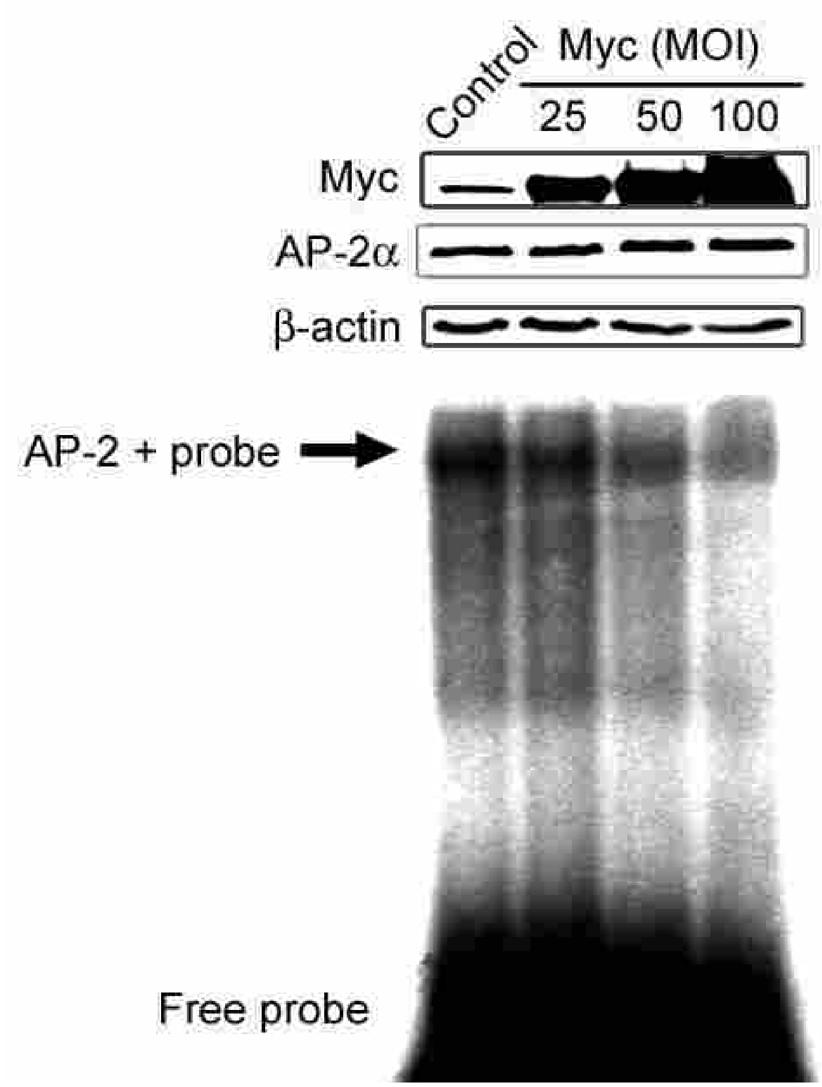

Supplemental Figure 2. Overexpression c-MYC protein caused decreasing of AP-2α DNA binding activity. HaCaT cells were infected with increasing MOI of Ad-c-MYC. 24 hours later, the nuclear protein was extracted and 40 μg was analyzed with western blot and 5 μg was analyzed with gel shift assay. AP-2α DNA binding activity decreased while c-MYC protein levels increased. AP-2α protein level also showed no significant increased after c-MYC infection.
